# EpCAM Immunotherapy versus Specific Targeted Delivery of Drugs

**DOI:** 10.3390/cancers10010019

**Published:** 2018-01-12

**Authors:** Joanna Macdonald, Justin Henri, Kislay Roy, Emma Hays, Michelle Bauer, Rakesh Naduvile Veedu, Normand Pouliot, Sarah Shigdar

**Affiliations:** 1 School of Medicine, Deakin University, Geelong, VIC 3128, Australia; jmmacd@deakin.edu.au (J.M.); jhenri@deakin.edu.au (J.H.); kislay.roy@deakin.edu.au (K.R.); ehays@deakin.edu.au (E.H.); mbauer@deakin.edu.au (M.B.); 2 Centre for Comparative Genomics, Murdoch University, Perth, WA 6150, Australia; r.veedu@murdoch.edu.au; 3 Perron Institute for Neurological and Translational Science, Perth, WA 6009, Australia; 4 Department of Pathology and University of Melbourne, Melbourne, VIC 3010, Australia; normand.pouliot@onjcri.org.au; 5 Sir Peter MacCallum Department of Oncology, University of Melbourne, Melbourne, VIC 3000, Australia; 6 Matrix Microenvironment and Metastasis Laboratory, Olivia Newton-John Cancer Research Institute, School of Cancer Medicine, La Trobe University, Heidelberg, VIC 3084, Australia; 7 Centre for Molecular and Medical Research, Deakin University, Geelong, VIC 3128, Australia

**Keywords:** antibody, aptamer, cancer, EpCAM, immunotherapy, therapeutics

## Abstract

The epithelial cell adhesion molecule (EpCAM), or CD326, was one of the first cancer associated biomarkers to be discovered. In the last forty years, this biomarker has been investigated for use in personalized cancer therapy, with the first monoclonal antibody, edrecolomab, being trialled in humans more than thirty years ago. Since then, several other monoclonal antibodies have been raised to EpCAM and tested in clinical trials. However, while monoclonal antibody therapy has been investigated against EpCAM for almost 40 years as primary or adjuvant therapy, it has not shown as much promise as initially heralded. In this review, we look at the reasons why and consider alternative targeting options, such as aptamers, to turn this almost ubiquitously expressed epithelial cancer biomarker into a viable target for future personalized therapy.

## 1. Introduction

Cancer therapy has progressed significantly in the last forty years. We have moved on, to an extent, from the delivery of non-specific drugs, such as taxanes, vinca alkaloids, anthracyclines, and others [1]. While these drugs all showed promise, they are often delivered at suboptimal levels due to their toxic side effects. However, a significant discovery in the 1970s by Kohler and Milstein led to the development of monoclonal antibodies [2]. Following this, functional screens were performed whereby cancer cells were injected into mice to generate monoclonal antibodies and a novel cancer specific cell surface antigen was discovered [3]. The epithelial cell adhesion molecule (EpCAM), or CD326, was one of the first cancer biomarkers that were discovered in the 1970s. This cell surface, type 1 transmembrane glycoprotein, is highly expressed in epithelial cancers, making it an ideal target for both diagnosis and therapy [4,5,6]. What makes EpCAM a very interesting molecule for targeted therapeutics though, is that it is localised to the basolateral membrane in normal epithelial tissue, but during the progression from normal cell to cancer cell, the expression pattern changes to an intense uniform membranous over-expression [4]. It is this intercellular sequestration that is thought to prevent therapeutic modalities targeting EpCAM from harming normal epithelial tissue as the intercellular boundaries are very dense and highly organised structures [7].

## 2. EpCAM as a Functional Molecule

EpCAM, as its name suggests, is involved in cell adhesion through Ca^2+^-independent homophilic cell-cell adhesion in cells that normally lack cell-cell interactions. However, EpCAM is not associated with any classical junctional structure or with any of the four major families of cell adhesion molecules (CAMs) [3]. In fact, while the other CAMs form tight connections between cells, EpCAM is relatively weak, and EpCAM expressing cells are only loosely interconnected. EpCAM has also been shown to modulate other CAM functions, and weakens E-Cadherin-mediated intercellular adhesion through reduced stability [3]. As well, contact inhibition and polarisation, which are typical epithelial features, are less strong in EpCAM-expressing cells. Finally, cell migration and motility are attenuated in murine knock-out cells, suggesting that EpCAM plays a role in cancer cell invasion and metastasis, and that, in cancer cells, where EpCAM is over-expressed, it acts as a negative regulator of adhesion [3,4,8]. It has also more recently been suggested to be an oncogene, and in most types of cancers, it is associated with poor prognosis [9,10]. Surprisingly though, increased EpCAM expression does not always suggest poor prognosis for patients, and it is very much context-driven due to the distinct biology that some subtypes of cancer have. For example, if EpCAM expression is considered for breast cancer, an over-expression suggests poorer prognosis, greater tumour size, lymph node involvement, tumour stage, and tumour grade [11,12]. However, if these cases are delineated into the distinct subtypes, EpCAM expression is associated with an unfavourable prognosis in the basal like triple negative breast cancers and luminal B subtypes, whereas in the human epidermal growth factor receptor 2 (HER2+) subtype, it is associated with a favourable prognosis [11]. Since the initial discovery of cancer associated biomarkers in the 1970s, several other biomarkers have been discovered and have been successfully targeted with therapeutics. So, why has EpCAM, which is expressed on the vast majority of cancers of epithelial origin, failed to deliver on the initial promises of forty years ago?

## 3. Targeted Therapeutics—The First Wave

EpCAM was first identified as a cancer-specific cell surface antigen during a functional screen in which cancer cells were injected into mice to generate antibodies [3,13]. The resultant hybridoma cells were reliable sources of monoclonal antibodies and given that they could bind to markers on cancer cells, these were considered as potential agents for targeted therapeutics. The first monoclonal antibody to be tested in clinical trials was the EpCAM-specific murine IgG2a antibody 17-1A produced in the ascites of mice and called edrecolomab. This antibody was first described in 1979, and tested in humans in the first trial several years later in patients with gastrointestinal adenocarcinoma [14]. Initial reports suggested that edrecolomab induced anti-tumour activity through the activation of an array of endogenous cytotoxic mechanisms, including both antibody-dependent cell-mediated cytotoxicity (ADCC) and complement-dependent cytotoxicity (CDC), as well as potentially inducing a host-idiotype antibody response [15,16]. However, while it showed some efficacy in earlier trials in resected colorectal cancer, its benefits could not be replicated in larger clinical trials [17,18]. It also led to significant adverse effects with both murine and chimeric clones of the antibody [13]. Given that EpCAM appeared to be a perfect target, however, further work was conducted to generate antibodies with moderate or high affinity to EpCAM, with the thinking that edrecolomab was inefficacious as it only had a low affinity for its target. With a low affinity, edrecolomab bound only to cells with high expression, but due to the heterogeneity of tumours, not all of the tumour cells overexpressed EpCAM to the extent that the antibody theoretically bound to them. The next two antibodies that were generated, 3622W94 and ING-1, were discontinued in clinical trials due to poor tolerability [13]. The issue with these were that, rather than sparing normal epithelial cells that have both a low target expression and an almost hidden expression due to EpCAM only being present on the basolateral membrane, they bound to all cells expressing EpCAM and caused acute pancreatitis. Having learnt from these experiences, the next antibody generated, Adecatumumab, had a moderate affinity for EpCAM, and spared the normal cells while targeting the tumour cells. This antibody has shown efficacy in clinical trials, and in one study of metastatic breast cancer, it reduced the number of metastases and lowered tumour progression. However, when patients were stratified based on EpCAM expression, it was patients who had a high expression of EpCAM that responded more favourably than those with a low expression of EpCAM on their cell surface [19]. However, what may be of more importance to the positive effects observed with Adecatumumab is that this antibody has been shown to have a small but significant inhibitory effect on cell metabolism, suggesting that ADCC and CDC only play small roles in the therapeutic effects of monoclonal antibodies [13].

## 4. How Do Monoclonal Antibodies Work as Therapeutics?

To understand why some of these therapeutic strategies did not work as expected, an understanding of ADCC and/or CDC is required. Antibodies mainly function as therapeutics in one of two ways, involving either effector cells of the immune system (ADCC) or independent of the immune cells, requiring circulating proteins of the complement system (CDC) [20]. CDC occurs via interactions with the Fc domain of the antibody. The Fc portion of the antibody binds via their Fab domain to the surface of a cell, but can also bind to C1q and cause the deposition of complement, which thus activates a cascade resulting in CDC [20,21]. For ADCC, at least as suggested through in vitro studies, an effector to target cell ratio of 25–50:1 is required [22]. ADCC is the result of Fc-gamma receptor (FcγR, CD16) mediated interaction with effector immune cells, such as natural killer (NK) cells, macrophages, and granulocytes. The binding of FcγR to the Fc domain induces the release of both granzyme and perforin from effector cells, which leads to target cell lysis and Fc-dependent tumour cell phagocytosis [23]. These effects are dependent on a number of factors, including the density of the antigen on the cell surface and the isotype of the antibody [21]. Indeed, the effects of ADCC or CDC can specifically depend on the affinity of the antibody to either FcγR or complement components, regardless of the affinity of the antibody to its target. 

In a perfect scenario, the monoclonal antibodies would be infused and they would travel directly to the tumour, where they bind to all the tumour cells, bind straight to either complement or CD16 positive cells, and cause cellular cytotoxicity. However, no targeted therapy immediately homes to the tumour, but needs to come into contact with the biomarker on the tumour cells during normal circulation and it can take up to 24 h for complete accumulation of antibodies in the tumour [24,25]. The vasculature of the tumour aids in ‘sucking in’ the targeting agent due to the leaky blood vessels. However, due to a lack of lymphatic drainage, backpressure can prevent some macromolecules from diffusing into the tumour, thus limiting the effective dose [26]. Antibodies with low affinity will bind to the first target they find, but may be released, allowing for them to penetrate further into the tumour than those with moderate to higher affinity, which would remain bound to the first cell surface marker they encounter [26]. Additionally, some studies have shown that metastatic lesions have higher expression levels of EpCAM than the primary tumours, suggesting that these antibodies might target these secondary lesions rather than showing any benefit to the primary tumour [12,27].

For these antibodies to work, there needs to be an adequate number of antibodies that are attached to the cells, and complement or CD16-positive cells in the vicinity of the tumour cells [24]. Given that in vitro assays for ADCC take 4–16 h incubation, it is likely that the shortest amount of time for this reaction to occur in vivo is several hours [21,23]. There is also the danger that due to limited penetration of drugs due to both the backpressure and the size of the antibody, there is limited penetration of the drugs through the tumour tissue [26,28,29]. This is more so the case when the antibody has a high affinity for its target and binds to the first receptor it encounters. However, studies have shown that ADCC effects do not necessarily correlate with antibody target expression, with one recent study assessing the ADCC effects of Trastuzumab and demonstrating that ADCC activity did not correlate with HER2 expression levels, with the cell line having a moderate expression of HER2 that had the highest ADCC activity [23].

Therefore, taking into account the other barriers in the way for the antibody to find the tumour, overcome the barriers of backpressure, and binding to and staying bound to the one tumour cell for long enough [20], an almost ‘perfect storm’ needs to occur for efficient tumour cell death. These factors could explain why, in xenograft models, less than 20% of the administered dose of antibodies typically interacts with the tumour [30]. However, this is not the only consideration for successful therapy. Previous studies with Cetuximab have demonstrated that tumor cell killing only occurs if the antibody remains bound to the outside of the cancer cell and is not internalised [31]). This is intuitive as the only way for effector cells or complement to bind to the antibody and come into contact with the tumour cell is if the antibody remains in contact with the cell within the microenvironment. However, studies have shown that EpCAM is rapidly internalised following ligands binding to the cell surface receptor [32,33]. So, the question is, of the antibodies that have been developed for therapeutic targeting of EpCAM, do they become internalised with the receptor, or remain within the vicinity of the cell when the receptor becomes internalised? Also, if they remain in the vicinity of the cell, how much does this hinder the ADCC or CDC process if they are continuously being released from the cell membrane?

**Figure 1:**
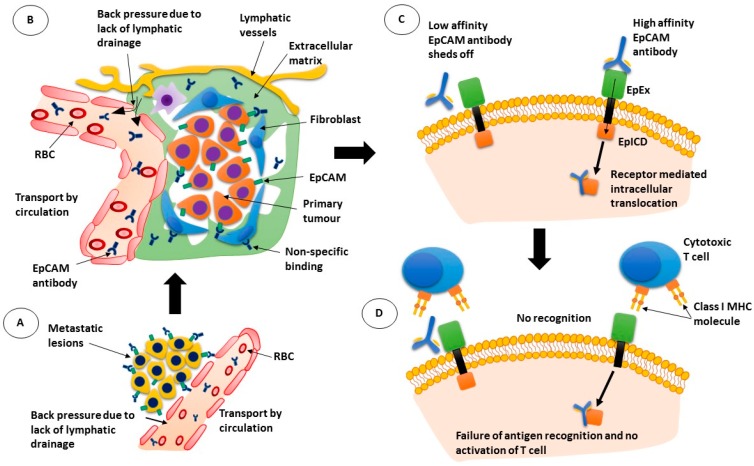
Mechanism of action of epithelial cell adhesion molecule (EpCAM) antibodies. (**A**) antibodies pass through the leaky blood vessel walls but are moved back into circulation due to back pressure; (**B**) while the EpCAM antibodies can bind to tumour cells, they can also bind to other cells non-specifically; (**C**) low affinity antibodies may not remain bound to their target while high affinity antibodies may be internalised with the receptor; (**D**) If the antibody is internalised, cytotoxic T cells are not activated. RBC: red blood cell; EpEx: extracellular domain of EpCAM; EplCD: intracellular domain of EpCAM; MHC: major histocompatibility complex.

Thus, as summarized in Figure 1, therefore, monoclonal antibodies need to combat a number of environmental factors before they can get close to the tumour cells. Once in their vicinity, they face a second set of challenges, such as maintaining contact with the cell, without being internalised, for a sufficient period of time to engage effector cells or complement. Additionally, there needs to be enough antibodies surrounding the cell to cause ADCC or CDC [24]. However, if the therapy is dependent on tumour infiltrating immune cells that participate in ADCC, there also needs to be a sufficient number of immune cells within the tumour microenvironment that can kill the tumour cells [24]. Further, this is assuming that there are no immune suppressor cells, such as regulatory T cell and myeloid-derived suppressor cells, which inhibit both NK cell function and ADCC activity [23,34]. 

## 5. Targeted Therapeutics—The Second Wave

Targeting EpCAM has shown promise, but due to the environmental issues, as well as the requirement for sufficient numbers of antibodies to surround each cancer cell in order to provoke an immune response from CD16-positive cells, the next wave of antibodies sought to harness the immune system by bringing the immune cells into close proximity with the tumour cells [35]. Catumaxomab is a trifunctional, bispecific antibody that binds to EpCAM, CD3-positive T cells, and uses its Fc region to bind CD16-positive cells. Catumaxomab combines the characteristics of classical monoclonal antibodies and bispecific molecules, and is known as a trifunctional, bispecific antibody (Triomab^®^). The interaction of different immune effector cells at the tumour site results in a complex immune reaction leading to the elimination of the tumour cells. This elimination was studied in vitro and consisted of T cell mediated lysis, cytotoxicity by released cytokines, phagocytosis, or ADCC [36,37]. Additionally, long-lasting anti-tumour immunity was demonstrated in a mouse tumour model. When it entered clinical trials, tumour cells were eliminated, potentially through an increase in cytokine release. Catumaxomab demonstrated clinical advantages and an acceptable toxicity profile for treatment of malignant ascites [37], as well as excellent dose-response profiles [38], and has been approved for clinical use following these successful trials [36]. 

Whereas, the Triomab^®^ format of antibodies are composed of two different full-size IgG-like half-antibodies [38], the bispecific T-cell engaging antibodies (BiTE^®^) are composed of two single chain variable fragments, each with a unique antigen specificity [35]. While catumaxomab was approved in the European Union in 2009 [39], the BiTE^®^ antibody targeting EpCAM has been slower to enter clinical trials. MT-110 has shown efficacy in several in vitro and in vivo studies, and has completed phase I dose escalation studies (NCT00635596), though no further studies are currently listed [35,36,40]. Very recent work has suggested that inhibiting immunosuppressive targets, such as indoleamine 2, 3-dioxygenase (IDO), may enhance the effects of MT110 [41], which also suggests the possibility of combining other similar immune inhibitors, such as programmed cell death protein-1 (PD-1) or cytotoxic T-lymphocyte-associated protein-4 (CTLA-4), for improved treatment outcomes. However, given the number of patients that have been recently diagnosed with autoimmune disorders following treatment with nivolumab (PD-1) or ipilimumab (CTLA-4), extra care needs to be taken when considering this [42]. 

For solid tumours, however, efficacy is very much dependent on the ability of the BiTE^®^ antibodies to penetrate into the tumour, as well as the presence of T-cells [35,36]. Given that these antibodies are smaller than full-length antibodies, there is hope that they will demonstrate continued promise for cancer patients. The one caveat to this is that they require functional T-cells, which may exhibit signs of exhaustion, and enhanced trafficking of additional functional T-cells to the tumour may be necessary to mitigate some of the non-responsiveness that is observed in patients [7,35,36].

## 6. Targeted Therapeutics—The Third Wave

In order to capitalise on the ability of antibodies to specifically target tumour cells, as well as to produce an efficacious therapeutic, the next step in developing EpCAM as a target for enhanced cytotoxicity has been to combine antibodies with cytotoxic drugs [1,29]. This would theoretically combine the best of both worlds, with the specificity of monoclonal antibodies and the cytotoxicity of small molecule drugs. Through the delivery of highly toxic drugs specifically into the cancer cells, it would not only increase the therapeutic window of the drug, but would also reduce some of the non-specific side effects observed in patients [1]. There have been a number of cytotoxic agents considered for attachment to antibodies that target either microtubules or DNA, with success being seen with compounds such as the maytansinoids, auristatins, calicheamicin, doxorubicin, paclitaxel derivatives, and topoisomerase I inhibitors [1,29,43]. What should be noted, however, is that unlike the first two examples that require external stimuli to kill the tumour cells, these antibody-drug conjugates need to be internalised so that the drug can have an effect [1,29]. As well, the binding affinity and number of antigen molecules on the cell surface may also affect the potency, as will the ratio of drug to antibody [1]. One study assessed the efficacy and concluded that a ratio of eight molecules of doxorubicin to antibody was necessary for curative efficacy [44].

One interesting antibody-drug conjugate involves the attachment of a mushroom toxin to an EpCAM antibody. Alpha-amanitin is the main component of the amatoxins and has been shown to inhibit DNA transcription and lead to apoptosis. This drug was attached to the EpCAM antibody, chiHEA125, through a glutarate linker with a ratio of between 4:1 and 8:1 drugs per antibody. The conjugation of the drug to the antibody had no effect on binding affinity, and demonstrated significant efficacy in an in vivo model of pancreatic carcinoma. As well as tumour volume decrease and a lack of tumour recurrence, increased apoptosis and decreased tumour cell proliferation was also observed [45]. A further drug that has been investigated for attachment to antibodies is the new class of DNA alkylating agents, indolinobenzodiazepine pseudodimers (IGN), which possess a single reactive imine group and are cytotoxic with high in vitro potency. Initial studies demonstrated troubling toxicity, possibly due to the DNA cross-linking feature of the IGN dimer, so further investigation of linking strategies were performed and the monoamine compound was found to alkylate DNA but not cross-link. While dose-dependent antigen-specific antitumour activity was demonstrated with the EpCAM-IGN, additional results are awaited [46]. 

Another innovation in this area is the use of photochemical internalisation (PCI), which is a drug delivery technology for local and light controlled cytosolic release of therapeutics [47]. This technology can spare healthy tissue as the cytotoxic effect is limited to the illuminated area. Lund and colleagues [47] linked saporin, a ribosome inactivating protein toxin, to an EpCAM antibody via a biotin-strepavidin linker, and was shown to selectively reduce cellular viability, proliferative capacity, and colony forming ability in vitro. Further studies are anticipated to demonstrate efficacy in in vivo models. 

While the size of antibodies has been recognised as a factor in ensuring sufficient drug concentration within the tumour, smaller peptides have been investigated for targeted drug delivery. Peptides were first proposed in 1992 as they are smaller than antibodies and are generally not cleared by the reticuloendothelial system [48]. They are also fairly easily synthesised and modified, and chemical modifications can prevent protein degradation [49]. However, while there are several hundred peptide candidates in the clinic and preclinical development, there are only a few that have been described for targeting EpCAM [49,50]. Designed Ankyrin Repeat Proteins (DARPins) are a novel class of non-immunoglobulin binding proteins thart rely on the modularity of ankyrins. Winkler and colleagues selected an EpCAM specific DARPin via ribosome display from a DARPin library [50]. This group also evolved the selection process to develop EpCAM-specific DARPins with picomolar affinity, and through the conjugation with a truncated form of the *Pseudomonas* exotoxin A, demonstrated enhanced cytotoxicity in vitro [51]. Though it only demonstrated a short half-life of 11 min, strong antitumour effects were observed in in vivo preclinical tumour models [52]. Macrocyclic peptides show higher affinity for their targets and possess higher resistance to degradation by proteases, as well as reduced hydrogen bonding, allowing for increased membrane penetrating potential [53]. A low nanomolar macrocyclic peptide has been generated to EpCAM using a random non-standard peptide-integrated discovery (RaPID) system [54]. This macrocyclic peptide was later attached to lipid nanoparticles and biodistribution and tumour targeting in vivo were investigated, though with limited success, possibly due to the short circulation time [55]. It will be interesting to see how these are further developed in the future.

## 7. Targeted Therapeutics—A New Hope?

One of the main issues with monoclonal antibodies is that there is insufficient penetration into the tumour tissue due to the size of the antibodies. A recent paper by Xiang and colleagues tested the ability of an EpCAM antibody to penetrate and be retained in vivo into a xenograft tumour [56]. In addition, they compared this to a ‘chemical’ antibody, or aptamer as they are also known, to determine which had the better biodistribution and pharmacokinetic profile. The chemical antibody has a higher accumulation in the tumour at both 3 and 24 h after intravenous administration, as well as a 4-fold better penetration, and more homogenous distribution [56], suggesting that these agents may represent a superior approach to targeted tumour therapeutics. These chemical antibodies, also known as aptamers, demonstrate the same binding kinetics as antibodies, but have several properties that make them more suited for the targeted delivery of therapeutics to solid tumours.

**Figure 2:**
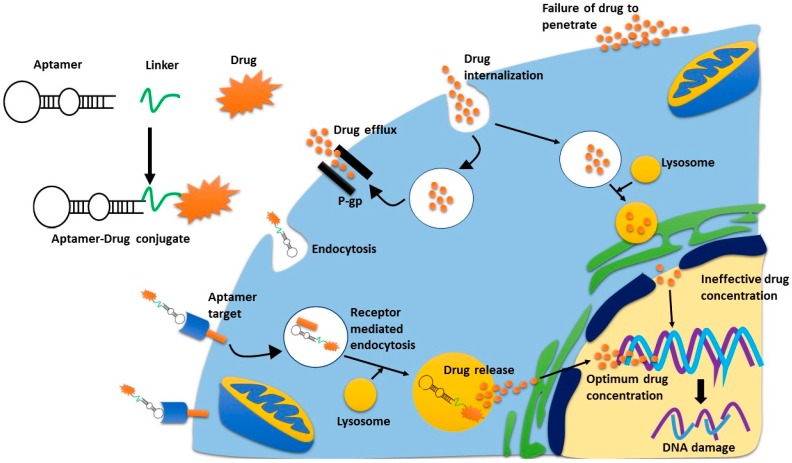
Receptor mediated endocytosis of cytotoxic agents. Aptamer-drug conjugates bind to their receptor, and are trafficked into the cell via receptor mediated endocytosis. Once in the lysosome, the drug is released from the aptamer and is then excreted from the lysosome where it can then move to the nucleus. If the drug is internalised non-specifically, drug-efflux pumps can pump the drugs outside the cell quickly, thus limiting their concentration inside the cell. This is the same process whereby antibody-drug conjugates can deliver a superior concentration of drug, as compared to non-specific cytotoxic drugs.

Aptamers are small, single stranded DNA or RNA oligonucleotides that bind to their targets in a similar manner to antibodies [57,58,59]. The ability of aptamers to bind to their target molecules is a result of their complex three-dimensional structures. Upon association with their target, aptamers form complex molecular architectures in which their target becomes an integral part of their structure via hydrogen bonding, van der Waals forces, and electrostatic interactions [58,60]. However, their other properties, such as small size, a lack of immunogenicity, and a ‘tunable’ half-life, mean that these aptamers can target solid tumours and act as agonists or antagonists, or deliver therapeutic cargoes while overcoming some of the barriers that prevent antibodies having a higher efficacy (Figure 2). Other notable advantages of aptamers over antibodies include the speed of generation (~2–8 weeks), ease of synthesis, no or very limited batch-to-batch variation, and their physical and thermal stability [58,60,61,62,63].

While there are aptamers that work by blocking the function of their target, such as the integrin DNA aptamer or VEGF aptamer [64,65] (and reviewed in [58,66]), the EpCAM aptamers have been developed to deliver cytotoxic agents [67]. Doxorubicin is one of the most widely used chemotherapeutic agents that is employed for a number of cancers, and has been previously linked to antibodies for targeted therapeutics [44]. The way in which doxorubicin exerts its effects and kills tumour cells is through intercalation into genomic DNA, thus disrupting replication and transcription. Fairly early on in the development of aptamers as therapeutic agents, it was realised that because aptamers form tertiary conformations with short double-stranded nucleic acid regions, they would require no or limited modifications for doxorubicin and an aptamer to form a physical complex [58,59,68]. This was firstly demonstrated by Bagalkot and colleagues in 2006 using an aptamer that targeted prostate specific membrane antigen, but was followed by a number of research groups [69,70,71,72]. Xiang et al. [67]. This aptamer was modified from the original aptamer developed to EpCAM [73] in that the double stranded stem was changed to G-C repeating DNA nucleotides to enhance the intercalation of doxorubicin. In this study, it was demonstrated that 2–3 molecules of doxorubicin intercalated into the stem of EpCAM aptamer, though if a higher efficacy is required, the double-stranded stem of the aptamer can be extended to allow for further drug attachment [67]. 

Given that aptamers are strings of nucleotides, there are many linkers that can be used to attach drugs to them with very limited modifications of the structure. The covalent coupling allows for the drug to be attached separately to the aptamer, meaning that it is unlikely to have any major effects on the specificity or sensitivity of the aptamer-drug conjugate. Additionally, the flexibility, pH sensitivity, and even length, can tailor-make the conjugate, allowing for several biochemical and biophysical parameters to be taken into account [68]. One drug conjugate attached via a cross-linker to an EpCAM aptamer was neocarzinostatin [74]. Neocarzinostatin is an anti-tumour antibiotic protein chromophore that has the ability to cleave double-stranded DNA [75]. In vitro studies demonstrated that this aptamer-drug conjugate was able to arrest cell cycle and promote apoptosis and necrosis [75,76]. 

There is still a lot of scope for the development of these aptamers for the delivery of other therapeutics. There have been a number of drugs conjugated to other aptamers which can easily be attached to the EpCAM aptamers. For example, a recent study by Yoon et al. incorporated both gemcitabine and 5-fluorouracil, or conjugated monomethyl auristatin E and derivative of maytansine 1 to an aptamer targeting pancreatic cancer [77]. These latter two agents are highly potent antimitotic drugs, but also show high toxicity, limiting their use as cytotoxic agents, though they have shown efficacy when conjugated to antibodies, as described previously. All four of these drugs were simply attached to the aptamer, either via enzymatic or chemical conjugation. In vitro studies so far have demonstrated a good efficacy of these agents [77], and it will be interesting to watch further development of these agents in aptamer-drug conjugates and see if other aptamers can demonstrate efficacy too. 

Aptamers are not without their challenges for therapeutic delivery, which is why so far there have only been a few commercialised aptamers [78], though there are now a number of aptamers entering clinical trials. The first issue to overcome is the stability of aptamers in biological fluids, with both their small size and nucleotide nature leading to renal excretion or nuclease degradation. However, there are a number of modifications that can prevent degradation, such as modified DNA and RNA bases, or 3’-capping with inverted thymidine bases [60]. The addition of drugs to the aptamer increases the molecular weight of the complex and could impede renal clearance, though it is very much dependent on the aptamer-drug conjugate having a molecular weight outside of the 30–50 kDa clearance range [79]. Finally, as aptamers are nucleotides, it is unsurprising that they have a net negative charge which can make them impenetrable to biological barriers [80]. However, if the aptamer is internalised following tight binding to its target, this electrostatic repulsive force effect is limited and appears to be alleviated by the binding affinity of the aptamer [62]. Indeed, in one study that directly compared the EpCAM antibody with the EpCAM aptamer in vivo, none of these factors affected the concentration of aptamer in the tumour, and the aptamer behaved superiorly to the antibody [56]. 

## 8. Conclusions

Antibodies were first described and tested as therapeutic agents almost 40 years ago and have shown efficacy against a number of biomarkers for the targeted treatment of cancer. EpCAM was one of the first biomarkers to be discovered, and there have been numerous clinical trials testing the efficacy of antibody therapy. While there have been a number of missteps, with high affinity antibodies or the lack of stratification of patients based on EpCAM tumour expression, there have now been some very successful trials in small cohorts of patients. However, in order to maximise EpCAM as a therapeutic target, different modalities are also required that have different mechanisms of action to monoclonal antibodies. Monoclonal antibodies are limited in their effects, either due to their lack of solid tumour penetration, or their need for active engagement of CD16-positive immune cells. Aptamers are smaller than antibodies, and thus can more effectively deliver drugs into the tumour. Numerous studies have demonstrated that, despite theoretical implications of rapid renal clearance, nuclease degradation, and electrostatic repulsion, aptamers are effective agents for the delivery of cytotoxic agents. These agents can either be used as singular agents, or given their different mechanism of action and suggested lack of drug-drug interaction, alongside antibodies to have a greater efficacy against solid tumours. EpCAM is a viable target for therapeutic delivery, but different mechanisms of action need to be considered in order for this almost ubiquitously expressed epithelial cancer biomarker to be an effective target for future personalised therapy.
